# Risk of Household Secondary Invasive Group A Streptococcal Infections After a Prophylaxis Policy Change

**DOI:** 10.1001/jamanetworkopen.2025.53168

**Published:** 2026-01-08

**Authors:** Brechje de Gier, Bart J. M. Vlaminckx, Annika van Roon, Mirjam J. Knol, Margreet J. M. te Wierik, Daan W. Notermans, Hester E. de Melker, Nina M. van Sorge

**Affiliations:** 1 Center for Infectious Disease Control, National Institute for Public Health and the Environment (RIVM), Bilthoven, the Netherlands; 2 Department of Medical Microbiology, University Medical Center Utrecht, Utrecht, the Netherlands; 3 Department of Medical Microbiology and Infection Prevention, Amsterdam University Medical Center location AMC, University of Amsterdam, Amsterdam Institute for Infection and Immunity, Amsterdam, the Netherlands; 4 Netherlands Reference Laboratory for Bacterial Meningitis, Amsterdam University Medical Center location AMC, Amsterdam, the Netherlands

## Abstract

**Importance:**

Household contacts of patients with invasive group A streptococcus (iGAS) disease have an increased risk of iGAS. In the Netherlands, the iGAS public health policy was changed in January 2023, offering antibiotic prophylaxis to household contacts of all patients with iGAS rather than only those presenting with necrotizing fasciitis or streptococcal toxic shock syndrome.

**Objective:**

To estimate risk of iGAS in the general population and among household and other contacts of primary patients with iGAS, before and after the policy change.

**Design, Setting, and Participants:**

This nationwide, population-based, open cohort study, linked population registry data with iGAS laboratory data for the study period (April 2022 to December 2024). The study population consisted of all persons included in the Dutch population registry at any time during the study period. The case definition was an iGAS isolate submitted to the Netherlands Reference Laboratory for Bacterial Meningitis, with disease onset in the study period.

**Exposure:**

For contacts of primary patients with iGAS, exposure risk period was defined as the 30 days after culture date of the index patient. Exposure under the new policy was defined as all person-time after January 20, 2023.

**Main Outcomes and Measures:**

Incidence rate ratios (IRR) of iGAS during the 30-day risk period compared with unexposed person-time were estimated. Secondary attack rates among household contacts were estimated with an odds ratio (OR) to compare attack rates before and after the policy change. Estimates were adjusted for age group, sex, household socioeconomic status, and year quarter.

**Results:**

A total of 19 006 247 persons (9 467 251 male [49.8%]; 6 308 794 [33.2%] aged 20-45 years) contributed 51 067 977 person-years to the analysis. A total of 3644 iGAS isolates from 3630 unique persons were included, of which 14 were household secondary cases. The IRR for household contacts during the risk period was 235.25 (95% CI, 94.35-586.59) before and 74.00 (95% CI, 35.17-155.71) after the policy change, compared with unexposed person-time. The secondary attack rate among household contacts was 0.219% (7 individuals) before and 0.047% (7 individuals) after the policy change (adjusted OR, 0.17; 95% CI, 0.03-0.83).

**Conclusions and Relevance:**

In this nationwide cohort study, there was a reduction in secondary iGAS risk among household contacts after implementation of an expanded antibiotic prophylaxis policy, which suggests that antibiotic prophylaxis for household contacts of patients with iGAS prevents secondary iGAS infection.

## Key Points

**Question:**

Was the Dutch public health policy change for invasive Group A Streptococcus (iGAS) disease, which expanded antibiotic prophylactic eligibility, associated with a decreased risk of secondary iGAS infection among household contacts?

**Findings:**

In this nationwide cohort study of 19 006 247 individuals, there was a significant 4.5-fold reduction in the iGAS secondary attack rate among household contacts after the policy change.

**Meaning:**

These findings suggest that antibiotic prophylaxis for household contacts of patients with iGAS prevents secondary iGAS infection.

## Teaser

This cohort study estimates the risk of invasive group A streptococcus (iGAS) before and after the implementation of a policy in the Netherlands that expanded antibiotic prophylaxis to household contacts of primary patients with iGAS.

## Introduction

*Streptococcus pyogenes* or Group A streptococcus (GAS) is a human-specific, Gram-positive bacterium that can be carried asymptomatically but also causes a large burden of noninvasive and invasive disease. Known risk factors for invasive GAS (iGAS) infections include young (0-5 years) or old (≥60 years) age, having underlying medical conditions, the peripartum period, and coinfections such as influenza or varicella.^1^ Although iGAS is uncommon, a surge of iGAS has been observed globally after lifting all societal restrictions related to the COVID-19 pandemic. iGAS has a high case fatality, estimated between 8% and 16%.^1^ Close contacts of a patient with iGAS have a greatly increased risk of developing iGAS, with estimates ranging between 800 to more than 5000 per 100 000 person-years at risk, compared with background incidences of 2 to 4 per 100 000 person-years.^1,2,3^ In the absence of effective vaccines, the only available public health measure to prevent iGAS is antibiotic prophylaxis for individuals after potential high-risk GAS exposure. There is currently no clear consensus about how to most effectively prevent secondary iGAS infection.

In 2008, 3 clinical presentations of iGAS became notifiable by law in the Netherlands: streptococcal toxic shock syndrome (STSS), necrotizing fasciitis, and puerperal sepsis. Household contacts of patients with STSS or necrotizing fasciitis were offered antibiotic prophylaxis to prevent secondary iGAS infection. In the Netherlands, a more than 2-fold increase in the annual number of notifiable iGAS infections was observed in 2022 compared with the annual average in the pre–COVID-19 pandemic years, leading to an estimated incidence of 10 per 100 000 per year.^4,5^ We have observed an absolute and relative increase of the globally disseminated *emm*1 clade M1_UK_, as well as the emergence of new variants of concern such as *emm*1.134 in 2022 to 2023, followed by an expansion of a previously rare *emm* type 3.93.^5,6^ A case-only analysis of the Dutch data showed that these 3 *emm* types more often clustered within households, suggesting an increased secondary attack rate.^7^ These observations prompted a change in the Dutch public health policy regarding antibiotic prophylaxis on January 19, 2023, where household contacts of all patients with iGAS were eligible for antibiotic prophylaxis to prevent secondary iGAS infection.^8^ To implement this policy, all iGAS infections (ie, all iGAS compatible disease presentations with *S pyogenes* detected in a normally sterile site) became notifiable by law. It was decided that a single daily dose of 500 mg of azithromycin for 3 days was the preferred prophylaxis regimen to optimize acceptability and adherence. However, because macrolide resistance is not rare among *S pyogenes* isolates,^4^ the prophylaxis can be changed based on the antibiotic susceptibility profile of the primary case. Alternative prophylactic regimes include either a 10-day course of clindamycin or oral penicillin in combination with 4 days of rifampicin. Both household and other close contacts are informed about their increased risk and are advised to seek medical care without delay in case of any symptoms consistent with GAS or iGAS infection, to enable early treatment.

The main aim of this retrospective population-based cohort study was to assess the impact of the policy change on the risk of secondary iGAS infection among household and other close contacts. We further describe the population incidence and risk factors for iGAS, and 30-day mortality after iGAS, in the Netherlands in the post–COVID-19 pandemic years (2022-2024).

## Methods

The National Institute for Public Health and the Environment (RIVM) Centre for Clinical Expertise verified whether this cohort study complied with the Dutch Law for Medical Research Involving Human Subjects (WMO) or with the European Union Clinical Trial Directive (2001/20/EC), and was of the opinion that review by an ethical research committee or institutional review board was not necessary by current national and European legislation (study number EPI-692). Informed consent was not required for this study because only pseudonymized microdata collected by Statistics Netherlands (CBS) under strict privacy safeguards were used, in accordance with the General Data Protection Regulation. Reporting follows the Strengthening the Reporting of Observational Studies in Epidemiology (STROBE) reporting guideline.

### Data Sources

The Netherlands Reference Laboratory for Bacterial Meningitis (NRLBM) has collected *S pyogenes* isolates for nationwide bacteriological surveillance of iGAS since April 2022. All medical microbiological laboratories in the Netherlands are requested to submit iGAS isolates to the NRLBM when cultured from a normally sterile site or from a nonsterile site in combination with a clinical presentation of iGAS. While submission is voluntary, the number of isolates received from unique patients during a time period when all iGAS was notifiable exceeded the number of iGAS notifications (2834 isolates vs 2492 notifications between February 2023 and December 2024). Also, the number of GAS isolates cultured from blood submitted to the NRLBM slightly exceeded the number of GAS blood cultures in the national antimicrobial susceptibility surveillance system ISIS-AR in 2023 (Boas Van der Putten, email communication, November 6, 2025). All iGAS isolates are *emm* typed or subtyped according to the Centers for Disease Control and Prevention protocol through polymerase chain reaction amplification and subsequent Sanger sequencing of the 180–base pair domain that contains the 50 hypervariable codons.^5^ Submitted isolates are accompanied by limited information regarding patient characteristics, including sex, birth date, and postal code. Through the Statistics Netherlands microdata system, isolates were linked to population registry data. Data on birth dates and sex were retrieved from the population registry.

### Definitions

An iGAS case was defined as a person with a linked iGAS isolate during the study period. A composite score of socioeconomic status (SES), based on income, wealth and occupation status, was available at the household level. Household compositions, with start and end dates, were used to identify household contacts and SES per participant. These households can be private or institutional (eg, long term care facilities for disabled or elderly care). Household contacts were defined as a person registered as belonging to the same household as a primary case at the iGAS disease onset date (the first known date of culture specimen or isolate receipt by the NRLBM). For clusters of coprimary cases (ie, contacts with the same index date), the primary and secondary case labels were assigned at random and one of the two was included as outcome event in the main analysis. For children born since 2022 and therefore not included in the person-network datasets, we assumed the nonhousehold relatives of their household contacts were also the nonhousehold relatives of the children. Nonhousehold relatives, colleagues, classmates, and neighbors were identified through the person-network datasets provided by Statistics Netherlands.^9,10,11,12^ Because these datasets were available for the period up to and including 2022, we assumed the contacts in 2022 were still their contacts in 2024. The period before the policy change was defined as April 1, 2022, to January 19, 2023, and compared with the period of January 20, 2023, to December 31, 2024, when the extended antibiotic prophylaxis policy was in effect.

### Statistical Analysis

An open cohort approach was used, where persons were included from April 1, 2022, or their birth date (whichever came last) and followed-up until December 31, 2024, their first iGAS episode, or death, whichever came first. When multiple isolates were received from the same person within 30 days, these were considered as belonging to the same episode. As primary outcome, the first iGAS episode per person was used. Persons with multiple iGAS episodes could, however, contribute to multiple exposure periods for their contacts. The primary exposure of interest was being a contact of a patient with iGAS during the 30-day risk period after the primary patient’s index date, under either the old or the new prophylaxis policy. The risk period was truncated in case of an event, therefore only the days leading up to the event of the contact are included in the person-time denominators. For secondary attack rate estimates, the number of contacts was the denominator.

Negative binomial regression was used to estimate incidence rate ratios (IRRs) of contacts during the risk period compared to the population background incidence. IRRs were compared before and after the policy change, and an interaction term between period and the exposure was used to test for significance (defined as a 2-sided *P* < .05). Logistic regression was used to compare secondary attack rates (SAR) among household contacts before and after the guideline change, and to estimate risk of 30-day mortality. All models were adjusted for age group, sex, calendar time (in year quarters), and household SES in quintiles (where quintile 1 represents the lowest SES). In the model comparing SAR among household contacts, SEs were adjusted for clustering by household. Analyses were performed in R version 4.5.0 (R project for Statistical Computing), using packages tidyverse, Epi, mgcv, lmtest, and multiwayvcov.

Results are based on calculations by the RIVM (project number 9892), using nonpublic microdata from Statistics Netherlands. As per the Statistics Netherlands policy, no numbers below 5 or estimates based on fewer than 5 events could be reported.

## Results

The Figure shows the number of *S pyogenes* isolates from patients with iGAS submitted to the NRLBM during the study period by *emm* type. During the 2022 to 2023 season, *emm*1.0 dominated, while *emm*3.93 dominated during the 2023 to 2024 season, as described previously.^5,6^ A total of 4215 iGAS isolate records were submitted to Statistics Netherlands, of which 3729 (88.5%) could be deterministically linked to unique persons in the population registry (eFigure 1 in Supplement 1). Linkage was significantly more often possible for ages 46 years and older, females, and isolates of *emm*4.0, but did not differ by policy period (eTable 1 in Supplement 1). Eighteen isolates were excluded because they were collected before the start of the study period, and a further 67 isolates were excluded when deduplicating isolates from the same person within 30 days. After exclusions, 3644 iGAS isolates from 3630 unique persons were included in the analysis.

**Figure.**
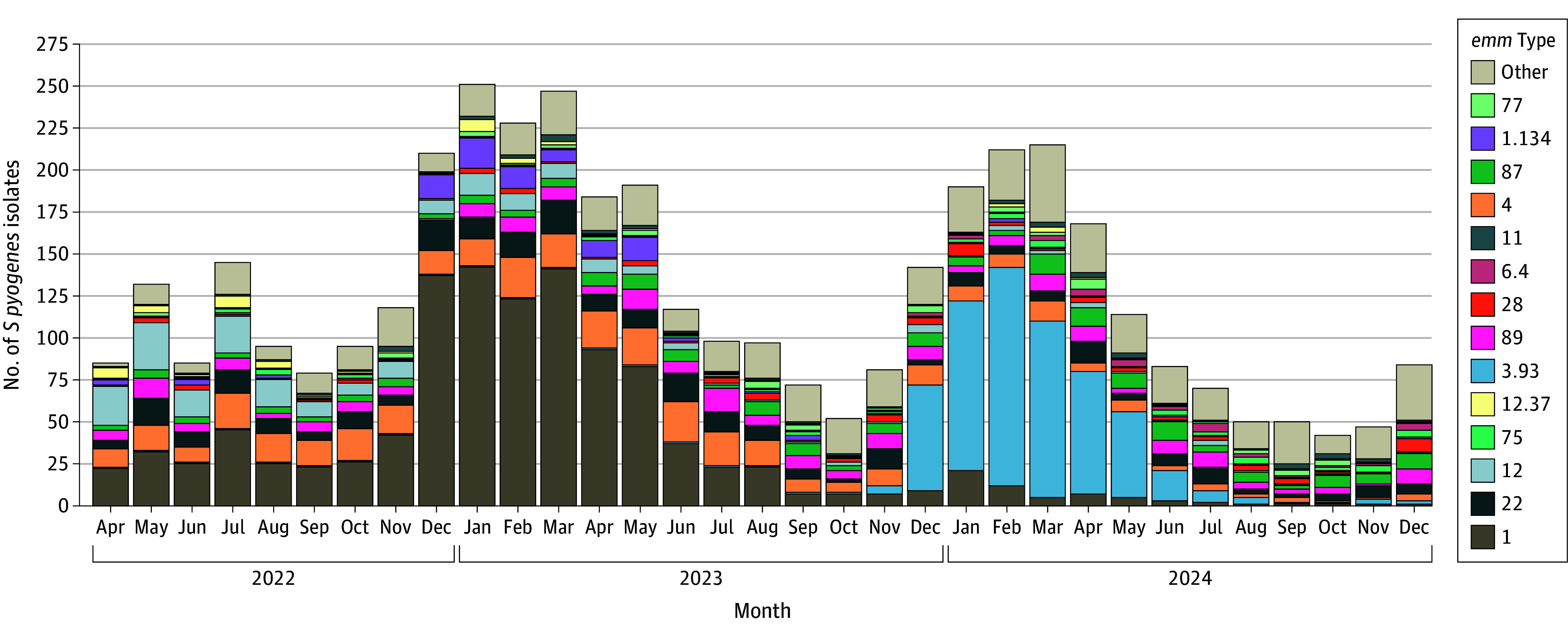
Number of *Streptococcus Pyogenes* Isolates Received and Typed by the Netherlands Reference Laboratory for Bacterial Meningitis During the Study Period by Month and *emm* Type (n = 4129)

A total of 19 006 247 persons contributed 51 067 977 person-years at any time during the study period. Of this population, 9 467 251 (49.8%) were registered as male and 6 308 794 (33.2%) were aged 20 to 45 years (eTable 2 in Supplement 1). Over the complete study period, 3609 of 3630 cases (99.4%) were primary iGAS cases (ie, cases not occurring during the risk period of a known contact), corresponding with a population background incidence of 7.07 per 100 000 person-years (Table 1). The incidence peaked in the first quarter of 2023 at 14.15 per 100 000 person-years (Table 1). Age groups 0 to 5 years (IRR, 1.98; 95% CI, 1.71-2.29) and 66 years and older (IRR, 2.13; 95% CI, 1.89-2.41) had a significantly higher incidence compared with age group 46 to 65 years. Persons registered as male had a significantly lower incidence compared with persons of female or unknown sex (IRR, 0.86; 95% CI, 0.79-0.94). This is likely related to the risk of puerperal fever or sepsis for women of childbearing age (ie, 20-45 years) (eFigure 2 in Supplement 1). Only the fourth quintile (second to highest) of household SES had a significantly higher incidence compared with the first quintile (IRR, 1.14; 95% CI, 1.00-1.30). Incidences by year quarter reflect the winter seasonality of iGAS.

**Table 1.** iGAS Events and Incidence by Exposures of Interest, April 2022 to December 2024, the Netherlands

Characteristic	Person-years, No.^a^	iGAS events, No.	Incidence per 100 000 person-years	IRR (95% CI)^b^
Exposure source				
None	51 033 734	3609	7.07	[Reference]
Household	1451	14	964.79	113.14 (65.49-195.46)
Nonhousehold relatives	7852	<5	NA	NA
Colleagues and classmates	11 178	<5	NA	NA
Neighbors	13 762	<5	NA	NA
Age group, y				
0-5	3 011 755	355	11.79	1.98 (1.71-2.29)
6-19	7 733 534	225	2.91	0.50 (0.42-0.59)
20-45	17 521 805	1128	6.44	1.05 (0.94-1.17)
46-65	13 381 750	790	5.90	[Reference]
≥66	9 419 133	1132	12.02	2.13 (1.89-2.41)
Sex				
Female or unknown	25 635 174	1952	7.61	[Reference]
Male	25 432 804	1678	6.60	0.86 (0.79-0.94)
Household socioeconomic status quintile				
1 (Lowest)	9 889 087	735	7.43	[Reference]
2	9 921 409	778	7.84	1.00 (0.88-1.13)
3	9 953 440	713	7.16	1.08 (0.95-1.22)
4	9 969 159	689	6.91	1.14 (1.00-1.30)
5	9 973 539	670	6.72	1.13 (0.99-1.29)
Unknown	1 361 345	45	3.31	NA
Year (quarter)				
2022 (2)	4 670 196	278	5.95	[Reference]
2022 (3)	4 671 408	279	5.97	0.98 (0.80-1.2)
2022 (4)	5 635 926	524	9.30	1.48 (1.24-1.78)
2023 (1)	3 605 008	510	14.15	2.16 (1.80-2.59)
2023 (2)	4 618 864	440	9.53	1.44 (1.20-1.74)
2023 (3)	4 670 868	236	5.05	0.82 (0.66-1.01)
2023 (4)	4 671 280	248	5.31	0.86 (0.70-1.06)
2024 (1)	4618692	556	12.04	1.84 (1.53-2.20)
2024 (2)	4 617 811	316	6.84	1.03 (0.85-1.26)
2024 (3)	4 669 237	153	3.28	0.52 (0.41-0.66)
2024 (4)	4 618 688	90	1.95	0.32 (0.24-0.41)

Abbreviations: iGAS, invasive group A streptococcus; IRR, incidence rate ratio; NA, not applicable.

^a^ Rounded to nearest integer.

^b^ Adjusted for age group, sex, household socioeconomic quintile, and year quarter.

In total, 14 secondary iGAS cases were identified among household contacts of primary patients with iGAS during the 30-day risk period, corresponding to an incidence of 964.79 per 100 000 person-years. The adjusted IRR for household contacts in the 30-day risk period compared with the population incidence was 113.14 (95% CI, 65.49-195.46) over the entire study period. For nonhousehold relatives, colleagues and classmates, and neighbors, the number of secondary cases was too low (<5) to present estimates according to the Statistics Netherlands policy.

Overall 30-day mortality was 10.2% (369 of 3630 individuals), and significantly lower for cases with *emm* type 4.0 compared with *emm* type 1.0 (17 of 362 cases [4.7%] vs 128 of 1030 cases [12.4%]; odds ratio [OR], 0.47; 95% CI, 0.26-0.78) (Table 2). Mortality was significantly lower for ages 0 to 45 years (0-5 years: OR, 0.49; 95% CI, 0.27-0.84; 6-19 years: OR, 0.33; 95% CI, 0.14-0.68; 20-45 years: OR, 0.29; 95% CI, 0.18-0.45) compared with 46 to 65 years, and significantly higher for ages 66 years and older (OR, 2.73; 95% CI, 2.04-3.70). No significant difference in mortality was observed between sexes, socioeconomic quintiles, or year quarters (Table 2).

**Table 2.** Thirty-Day Mortality of iGAS Cases and Mortality Risk by *emm* Type and Other Characteristics^a^

Characteristic	iGAS cases, No.	30-d Mortality, No. (%)	30-d Mortality, OR (95% CI)
Overall	3630	369 (10.2)	NA
*emm* Type			
1.0	1030	128 (12.4)	1 [Reference]
12.0	192	25 (13.0)	0.99 (0.58-1.65)
22.0	266	21 (7.9)	0.73 (0.42-1.22)
3.93	523	72 (13.8)	1.04 (0.65-1.65)
4.0	362	17 (4.7)	0.47 (0.26-0.78)
87.0	173	12 (6.9)	0.62 (0.31-1.17)
89.0	187	18 (9.6)	0.75 (0.40-1.32)
Other *emm* types	897	76 (8.5)	0.66 (0.46-0.93)
Age group, y			
0-5	355	16 (4.5)	0.49 (0.27-0.84)
6-19	226	7 (3.1)	0.33 (0.14-0.68)
20-45	1127	27 (2.4)	0.29 (0.18-0.45)
46-65	792	71 (9.0)	1 [Reference]
≥66	1130	248 (21.9)	2.73 (2.04-3.70)
Sex			
Female or unknown	1952	165 (8.5)	1 [Reference]
Male	1678	204 (12.2)	1.16 (0.92-1.46)
Household socioeconomic status quintile			
1 (Lowest)	735	94 (12.8)	1 [Reference]
2	778	116 (14.9)	1.10 (0.81-1.50)
3	713	73 (10.2)	1.05 (0.75-1.49)
4	689	48 (7.0)	0.95 (0.64-1.39)
5	670	32 (4.8)	0.83 (0.52-1.29)
Unknown	45	6 (13.3)	NA
Year (quarter)			
2022 (2)	278	20 (7.2)	1 [Reference]
2022 (3)	279	30 (10.8)	1.58 (0.85-2.99)
2022 (4)	524	66 (12.6)	1.51 (0.87-2.72)
2023 (1)	510	51 (10.0)	1.16 (0.66-2.13)
2023 (2)	440	36 (8.2)	0.96 (0.53-1.80)
2023 (3)	236	17 (7.2)	1.15 (0.56-2.35)
2023 (4)	248	23 (9.3)	1.23 (0.62-2.44)
2024 (1)	556	70 (12.6)	1.43 (0.78-2.71)
2024 (2)	316	36 (11.4)	1.21 (0.64-2.36)
2024 (3)	153	13 (8.5)	1.11 (0.50-2.42)
2024 (4)	90	7 (7.8)	1.02 (0.37-2.55)

Abbreviations: iGAS, invasive group A streptococcus; NA, not applicable; OR, odds ratio.

^a^ Study period ranged from April 1, 2022, to December 31, 2024. ORs were adjusted for age group, sex, household socioeconomic quintile, and year quarter.

### Outcomes of Policy Change

In the period before the policy change (April 1, 2022, to January 19, 2023), 7 secondary cases were identified among 3195 household contacts, resulting in an IRR of 235.25 (95% CI, 94.35-586.59) compared with the population incidence (Table 3). After the policy change on January 20, 2023, there was a significant reduction in the IRR to 74.00 (95% CI, 35.17-155.71) (*P* for interaction = .02), also based on 7 secondary cases among 14 974 household contacts. The incidence per 100 000 person-years decreased from 2995 before the policy change to 575 after the policy change. The population background incidence and the median number of household contacts per primary case did not differ between the 2 periods. Secondary attack rates among household contacts were 0.219% (7 individuals) before and 0.047% (7 individuals) after the guideline change (adjusted OR, 0.17; 95% CI, 0.03-0.83).

**Table 3.** iGAS Infections in Households in the Netherlands, Stratified by Periods With the Old and New Policy

Outcome	Old policy (April 1, 2022, to January 19, 2023)	New policy (January 20, 2023, to December 31, 2024)
Primary iGAS cases in multiperson households, No.	927	2213
Household contacts, No.	3195	14 974
Household contacts per case, median (IQR)	2 (1-3)	2 (1-3)
Secondary iGAS cases among household contacts, No.	7	7
Secondary attack rate, %	0.219	0.047
Person-years household contacts^a^	234	1217
Incidence among household contacts per 100 000 person-years	2995	575
Background population incidence per 100 000 person-years	7.17	7.03
IRR compared to unexposed (95% CI)^b^	235.25 (94.35-586.59)	74.00 (35.17-155.71)

Abbreviations: iGAS, invasive group A streptococcus; IRR, incidence rate ratio.

^a^ Rounded to nearest integer.

^b^ Adjusted for age group, gender, household socioeconomic quintile and year-quarter.

## Discussion

In this cohort study, we observed a significant reduction in risk of secondary iGAS infection for household contacts after a policy change that extended antibiotic prophylaxis eligibility to household contacts of all patients with iGAS rather than only household contacts of primary cases with STSS or necrotizing fasciitis. This reduction was visible both as a 4.5-fold reduction in the SAR, as well as a 3-fold difference in the IRR, where the person-time denominator was truncated in case of an event. Data were not available on the actual prescription or uptake of antibiotic prophylaxis; therefore, we could not estimate direct effectiveness of the prophylaxis against secondary iGAS infection.

Based on a 2023 study in the Netherlands,^13^ we know that there was a high overall acceptance of antibiotic prophylaxis with an uptake of 95% among eligible contacts. Of these contacts, 4.4% developed symptoms consistent with mild GAS infection, but no secondary iGAS infection was observed.^13^ A recent study by Birck et al^14^ linked prophylaxis prescription data, and found similar GAS infection rates among family contacts of primary iGAS cases who did and did not receive antibiotic prophylaxis. According to a recent systematic review,^15^ there is 1 previous study reporting GAS risk reduction after antibiotic prophylaxis, but this was during an outbreak among persons experiencing homelessness. Because there is currently no general consensus as how to best prevent secondary iGAS infections in the general population, we believe our study contributes valuable insights on the benefits of antibiotic prophylaxis to prevent iGAS in high-exposure settings.

In 2020, Adebanjo et al^2^ summarized the available evidence on iGAS incidence among household members of primary iGAS cases. The 2 largest previous studies found 1240 and 4520 iGAS cases per 100 000 person-years in the 30-day risk period among close contacts.^2,3^ Our incidence estimates for household contacts in this risk period, of 2995 before and 575 per 100 000 person-years after the policy change, are in line with this order of magnitude.

After the COVID-19 pandemic, many countries reported an increase in iGAS incidence in 2022 to 2023, including the Netherlands.^4^ Here, we report a population iGAS incidence of 7.07 per 100 000 person-years during 2022 to 2024, peaking in early 2023 at 14.15 per 100 000 person-years, which is roughly in line with published population incidences covering this period.^16,17^ A Danish study^18^ reported an incidence of 6.6 per 100 000 in children aged 0 to 17 years in 2022 to 2023, with the majority younger than 5 years, consistent with the incidence in children in our study (Table 1).

Surprisingly, we did not observe an association of iGAS risk with low household SES. Concordantly, 30-day mortality did not differ significantly by SES. Deprivation has been repeatedly shown to be an important risk factor for iGAS.^1,16,19,20^ Likely, our categorization of SES was not sensitive enough to capture the vulnerable populations most at risk for iGAS, such as people who inject drugs and people experiencing homelessness.

### Limitations

Our study has limitations, mainly affecting the estimated impact of the policy change on secondary iGAS incidence. The patient data submitted with the iGAS isolate did not allow us to distinguish between iGAS disease presentations for which prophylaxis was already indicated in 2022 (ie, STSS and necrotizing fasciitis) vs newly notifiable iGAS. Especially for clusters with isolates cultured just around the date of the policy change, it is not clear if the contacts were already offered prophylaxis. One cluster had an index date before the policy change but secondary iGAS infection after. We decided to categorize this as a cluster under the old policy. Due to the low number of secondary cases, this decision impacted our results, increasing the SAR under the old policy. Also, the proportion of secondary cases occurring too soon after the primary case to receive prophylaxis may have differed between the 2 periods, which impacts the preventable fraction of secondary iGAS infections. The low number of (secondary) cases limited the power of our study and precluded analyses among other types of close contacts such as nonhousehold family members. Further, because dominant *emm* types also differed between the periods, it is not possible to fully disentangle effects of the policy change and differences in transmissibility or virulence of circulating *emm* types, despite adjusting estimates for calendar time. Lastly, the fact that 11.5% of submitted isolates could not be deterministically linked to a unique person in the population registry presents an important limitation, resulting in a lower incidence estimate. Because linkage was based on postal code, birth date, and sex, it is possible that twins and persons in large institutional households were overrepresented in the nonlinked isolates. This might have resulted in an underestimation of the number of secondary household cases for both periods. While the median and IQR of household contacts per case were identical between the periods, the averages are very different, indicating a skewedness due to large institutional households included in the period with the new policy. Despite the adjustment for age group, this may have resulted in a more fragile population at risk included in the new policy period. On the other hand, the closeness of the contacts is likely to be less in such institutional households. The data did not allow us to distinguish between wards or rooms within institutional households, and the intensity of contact between household members may vary significantly.

The number of secondary iGAS cases was insufficient for a thorough analysis of host risk factors among the close contacts. Some countries, such as the UK, base prophylaxis eligibility not only on being a close contact to an index case but also on factors such as age, pregnancy, and viral infections.^1^ In the Netherlands, the deliberate choice was made not to make such a distinction, for 2 reasons: to prevent ongoing household transmission through a third (nonrisk group) household member and to enhance feasibility of timely prophylaxis by removing the need to assess risk factors.

## Conclusions

In this nationwide cohort study, we observed a substantial reduction in the risk of secondary iGAS infection among household contacts after the Dutch iGAS antibiotic prophylaxis policy change. However, the overwhelming majority of iGAS occurs as sporadic cases. Therefore, while antibiotic prophylaxis likely prevents a significant disease burden in contacts at high risk, the impact on overall iGAS incidence can only be very modest.

## Notes

Supplement 1. eFigure 1. Flowchart of inclusions of iGAS isolates, the Netherlands, for the study period 1 April 2022-31 December 2024 eTable 1. Linkage of isolate data to population registry by age group, gender, policy period and emm type, 1 April 2022-31 December 2024, the Netherlands eTable 2. Characteristics of the study population eFigure 2. Population iGAS incidence per 100,000 by age group and sex, 1 April 2022- 31 December 2024, the Netherlands

Supplement 2. Data Sharing Statement

## References

[1] Watts V, Usdin M, Mearkle R, ; Working Group for the UK Guidelines for the Management of Contacts of Invasive Group A streptococcus (iGAS) Infection in Community Settings. Antibiotic chemoprophylaxis for close contacts of invasive group A streptococcus in community settings: evidence review. J Infect. 2025;90(4):106468. doi:10.1016/j.jinf.2025.106468 40089213

[2] Adebanjo T, Apostol M, Alden N, . Evaluating household transmission of invasive group A streptococcus disease in the United States using population-based surveillance data, 2013-2016. Clin Infect Dis. 2020;70(7):1478-1481. doi:10.1093/cid/ciz716 31408094 PMC8935355

[3] Mearkle R, Saavedra-Campos M, Lamagni T, . Household transmission of invasive group A Streptococcus infections in England: a population-based study, 2009, 2011 to 2013. Euro Surveill. 2017;22(19):30532. doi:10.2807/1560-7917.ES.2017.22.19.30532 28537550 PMC5476984

[4] de Gier B, Marchal N, de Beer-Schuurman I, ; ISIS-AR Study Group; GAS Study Group. Increase in invasive group A streptococcal (*Streptococcus pyogenes*) infections (iGAS) in young children in the Netherlands, 2022. Euro Surveill. 2023;28(1):2200941. doi:10.2807/1560-7917.ES.2023.28.1.2200941 36695447 PMC9817208

[5] Rümke LW, Davies MA, Vestjens SMT, . Nationwide upsurge in invasive disease in the context of longitudinal surveillance of carriage and invasive *Streptococcus pyogenes* 2009-2023, the Netherlands: a molecular epidemiological study. J Clin Microbiol. 2024;62(10):e0076624. doi:10.1128/jcm.00766-24 39194268 PMC11481533

[6] Davies MA, de Gier B, Guy RL, . Streptococcus pyogenes emm Type 3.93 emergence, the Netherlands and England. Emerg Infect Dis. 2025;31(2):229-236. doi:10.3201/eid3102.240880 39983683 PMC11845126

[7] de Gier B, te Wierik M, Mujakovic S, Notermans D, de Melker H, van Sorge N. Invasive group A Streptococcal infections-increase and response 2022-2023. *Netherlands J of Med Microbiol*. 2023;(4):145-150 10.1001/jamanetworkopen.2025.53168 PMC12784225 41505128

[8] Rijksinstituut voor Volksgezondheid en Milieu. LCI-richtlijn groep A-streptokokkeninfectie. Published December 8, 2023. Accessed November 25, 2025. https://lci.rivm.nl/richtlijnen/groep-streptokokkeninfectie.

[9] Statistics Netherlands (CBS). Colleganetwerktab: collegarelaties. Published April 30, 2025. Accessed November 25, 2025. https://www.cbs.nl/nl-nl/onze-diensten/maatwerk-en-microdata/microdata-zelf-onderzoek-doen/microdatabestanden/colleganetwerktab-collegarelaties

[10] Statistics Netherlands (CBS). Burennetwerktab: buren- en buurtgenotenrelaties. Published April 30, 2025. Accessed November 25, 2025. https://www.cbs.nl/nl-nl/onze-diensten/maatwerk-en-microdata/microdata-zelf-onderzoek-doen/microdatabestanden/burennetwerktab-buren-en-buurtgenotenrelaties

[11] Statistics Netherlands (CBS). Familienetwerktab: familierelaties. Published April 30, 2025. Accessed November 25, 2025. https://www.cbs.nl/nl-nl/onze-diensten/maatwerk-en-microdata/microdata-zelf-onderzoek-doen/microdatabestanden/familienetwerktab-familierelaties

[12] Statistics Netherlands (CBS). Klasgenotennetwerktab: klasgenotenrelaties. Published April 30, 2025. Accessed November 25, 2025. https://www.cbs.nl/nl-nl/onze-diensten/maatwerk-en-microdata/microdata-zelf-onderzoek-doen/microdatabestanden/klasgenotennetwerktab-klasgenotenrelaties

[13] Mujakovic S, Ter Waarbeek HLG, Van den Boogaard J. Attitude towards antibiotic prophylaxis among household contacts of patients with invasive Group A Streptococcal disease and its impact on contacts’ iGAS incidence in the Netherlands. Poster presented at: European Scientific Conference on Applied Infectious Disease Epidemiology (ESCAIDE) 2023; November 23, 2023; Barcelona, Spain. Accessed November 25, 2025. https://www.escaide.eu/en/publications-data/escaide-2023-abstract-book

[14] Birck MG, Moura CS, Winthrop KL, Machado MAA, Neville A, Bernatsky S. Choosing wisely: effectiveness and safety of antibiotic prophylaxis in close contacts of invasive group A streptococci infection. Clin Infect Dis. 2025;80(2):314-315. doi:10.1093/cid/ciae524 39589137 PMC11848273

[15] Khan F, Bai Z, Kelly S, . Effectiveness and safety of antibiotic prophylaxis for persons exposed to cases of invasive group a streptococcal disease: a systematic review. Open Forum Infect Dis. 2022;9(8):ofac244. doi:10.1093/ofid/ofac244 36046698 PMC9424867

[16] Gregory CJ, Okaro JO, Reingold A, . Invasive group A streptococcal infections in 10 US states. JAMA. 2025;333(17):1498-1507. doi:10.1001/jama.2025.0910 40193120 PMC11976646

[17] Valcarcel Salamanca B, Cyr PR, Bentdal YE, . Increase in invasive group A streptococcal infections (iGAS) in children and older adults, Norway, 2022 to 2024. Euro Surveill. 2024;29(20):2400242. doi:10.2807/1560-7917.ES.2024.29.20.2400242 38757285 PMC11100296

[18] Nygaard U, Hartling UB, Munkstrup C, . Invasive group A streptococcal infections in children and adolescents in Denmark during 2022-23 compared with 2016-17 to 2021-22: a nationwide, multicentre, population-based cohort study. Lancet Child Adolesc Health. 2024;8(2):112-121. doi:10.1016/S2352-4642(23)00295-X 38103567

[19] Ammar S, Anglemyer A, Bennett J, . Post-pandemic increase in invasive group A strep infections in New Zealand. J Infect Public Health. 2024;17(11):102545. doi:10.1016/j.jiph.2024.102545 39303459

[20] Zangarini L, Martiny D, Miendje Deyi VY, . Incidence and clinical and microbiological features of invasive and probable invasive streptococcal group A infections in children and adults in the Brussels—capital region, 2005-2020. Eur J Clin Microbiol Infect Dis. 2023;42(5):555-567. doi:10.1007/s10096-023-04568-y 36881216 PMC9989989

