# Supplementary material for: Risk of Household Secondary Invasive Group A Streptococcal Infections After a Prophylaxis Policy Change
Source: JAMA Netw Open. 2026 Jan 8;9(1):e2553168. doi: 10.1001/jamanetworkopen.2025.53168 (PMC12784225; doi:10.1001/jamanetworkopen.2025.53168)
Supplement: Supplement 1. — eFigure 1. Flowchart of inclusions of iGAS isolates, the Netherlands, for the study period 1 April 2022-31 December 2024 eTable 1. Linkage of isolate data to population registry by age group, gender, policy period and emm type, 1 April 2022-31 December 2024, the Netherlands eTable 2. Characteristics of the study population eFigure 2. Population iGAS incidence per 100,000 by age group and sex, 1 April 2022- 31 December 2024, the Netherlands [file jamanetwopen-e2553168-s001.pdf]

Supplemental Online Content

de Gier B, Vlaminckx BJM, van Roon A, et al. Risk of Household Secondary Invasive Group A Streptococcal Infections After a Prophylaxis Policy Change . *JAMA Netw. Open*. 2026;9(1):e2553168. doi: 10.1001/jamanetworkopen.2025.53168

eFigure 1. Flowchart of inclusions of iGAS isolates, the Netherlands, for the study period 1 April 2022-31 December 2024

eTable 1. Linkage of isolate data to population registry by age group, gender, policy period and emm type, 1 April 2022-31 December 2024, the Netherlands

eTable 2. Characteristics of the study population

eFigure 2. Population iGAS incidence per 100,000 by age group and sex, 1 April 2022- 31 December 2024, the Netherlands

This supplemental material has been provided by the authors to give readers additional information about their work.

© 2026 de Gier et al. *JAMA Network Open*.

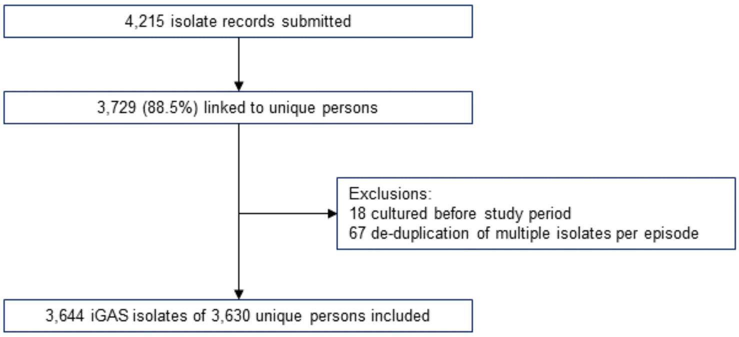

eFigure 1. Flowchart of inclusions of iGAS isolates, the Netherlands, for the study period 1 April 2022-31 December 2024.

eTable 1. Linkage of isolate data to population registry by age group, gender, policy period and *emm* type, 1 April 2022-31 December 2024, the Netherlands.

Variable	Level	Not linked	Linked	Percentage linked	OR (95% confidence interval)
Age group	0-5	52	375	87.8	[ref]
	6-19	26	243	90.3	1.44 (0.86-2.41)
	20-45	152	1127	88.1	1.05 (0.73-1.47)
	46-65	61	807	93.0	2.08 (1.39-3.1)
	66+	65	1160	94.7	2.77 (1.87-4.09)
Sex	Male	182	1713	90.4	[ref]
	Female or unknown	174	1999	92.0	1.33 (1.06-1.67)
Policy	New (20 January 2023- 31 December 2024)	249	2606	91.3	[ref]
	Old (1 April 2022- 19 January 2023)	107	1106	91.2	1.04 (0.81-1.35)
emm type	1.0 Cluster A-C3	103	1051	91.1	[ref]
	1.134 Cluster A-C3	6	102	94.4	1.6 (0.74-4.18)
	12.0 Cluster A-C4	20	201	91.0	1.06 (0.65-1.81)
	22.0 Cluster E4	23	271	92.2	1.27 (0.80-2.09)
	3.93 Cluster A-C5	36	538	93.7	1.42 (0.95-2.16)
	4.0 Cluster E1	22	371	94.4	1.90 (1.20-3.14)
	87.0 Cluster E3	13	174	93.0	1.36 (0.77-2.61)
	89.0 Cluster E4	22	192	89.7	0.86 (0.54-1.44)
	Other *emm* type	111	812	88.0	0.71 (0.53-0.95)

© 2026 de Gier et al. *JAMA Network Open*.

eTable 2. Characteristics of the study population.

		N (%)
Total		19,006,247
Age group (years) at the start of follow-up	0-5	1,559,629 (8.2)
	6-19	2,906,149 (15.3)
	20-45	6,308,794 (33.2)
	46-65	4,888,424 (25.7)
	66+	3,343,251 (17.6)
Sex	Female or unknown	9,538,996 (50.2)
	Male	9,467,251 (49.8)
Household socioeconomic quintile	1	3,697,898 (20.0)
	2	3,697,894 (20.0)
	3	3,697,536 (20.0)
	4	3,697,587 (20.0)
	5	3,697,546 (20.0)

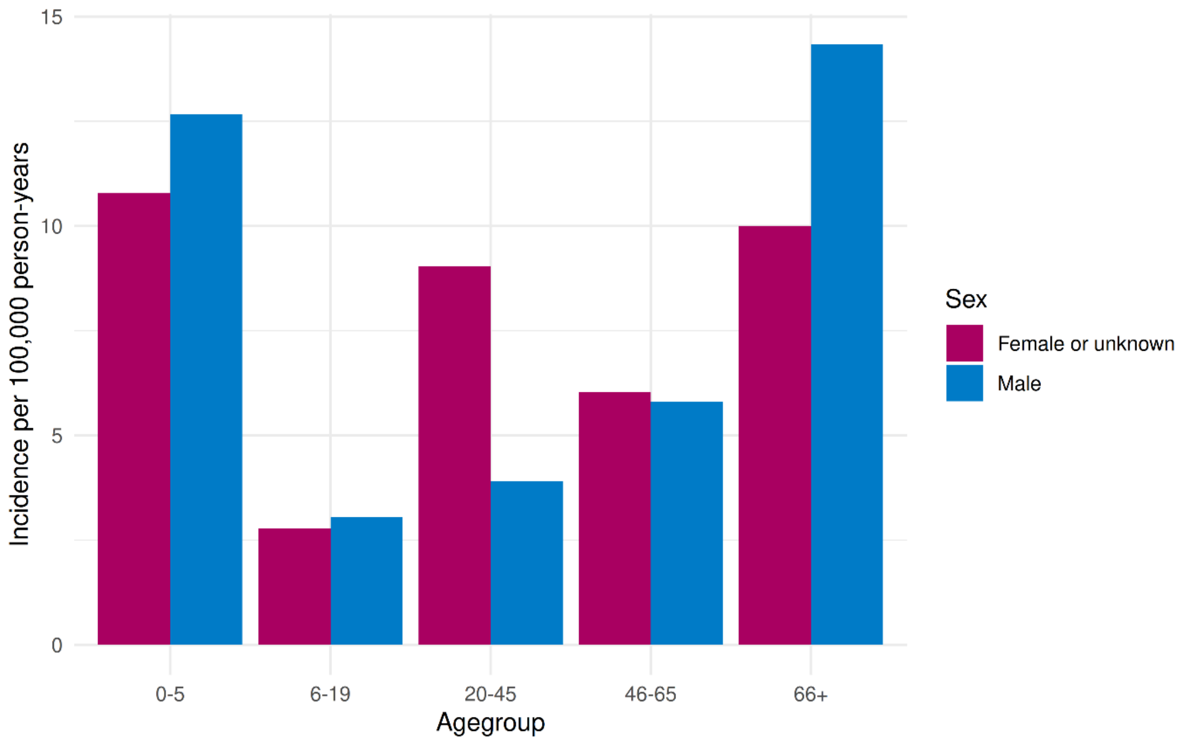

eFigure 2. Population iGAS incidence per 100,000 by age group and sex, 1 April 2022- 31 December 2024, the Netherlands.

© 2026 de Gier et al. *JAMA Network Open*.
