# Supplementary material for: Risk of Household Secondary Invasive Group A Streptococcal Infections After a Prophylaxis Policy Change
Source: JAMA Netw Open. 2026 Jan 8;9(1):e2553168. doi: 10.1001/jamanetworkopen.2025.53168 (PMC12784225; doi:10.1001/jamanetworkopen.2025.53168)
Supplement: Supplement 2. — Data Sharing Statement [file jamanetwopen-e2553168-s002.pdf]

Data Sharing Statement

de Gier. Risk of Household Secondary Invasive Group A Streptococcal Infections After a Prophylaxis Policy Change. *JAMA Netw Open*. Published January 08, 2026. doi:10.1001/jamanetworkopen.2025.53168

Data
Data available: No

Additional Information
Explanation for why data not available: This study was performed using non-public microdata from Statistics Netherlands and laboratory data from the Netherlands Reference Laboratory for Bacterial Meningitis (Amsterdam UMC, Amsterdam, Netherlands). These data cannot be shared by the authors.
